# Prognostic significance of additional histologic features for subclassification of pathological T3 colon cancer

**DOI:** 10.1007/s10147-022-02192-y

**Published:** 2022-06-18

**Authors:** Lorenzo Macchi, Quoc Riccardo Bao, Laura Albertoni, Matteo Fassan, Valentina Chiminazzo, Marco Scarpa, Gaya Spolverato, Salvatore Pucciarelli

**Affiliations:** 1 General Surgery 3, Department of Surgical, Oncological, and Gastroenterological Sciences, University of Padova, Via Giustiniani 2, 35128 Padua, Italy; 2 Surgical Pathology and Cytopathology Unit, Department of Medicine-DIMED, University of Padova, Padua, Italy; 3 Veneto Institute of Oncology (I.O.V. IRCSS), Padua, Italy; 4 Unit of Biostatistics, Epidemiology and Public Health, Department of Cardiac, Thoracic, Vascular Sciences and Public Health, University of Padova, Padua, Italy

**Keywords:** Colon cancer, Depth of infiltration, Elastic lamina invasion

## Abstract

**Background:**

Additional histologic features of T3 colon cancer, such as tumour depth invasion beyond muscularis propria and elastic lamina invasion (ELI), have taken interest for a more accurate staging.

**Methods:**

Patients with pT3 and pT4a (control group) colon adenocarcinoma were retrospectively collected from our institutional database. The study group was divided according to depth of tumour invasion < 5 mm and ≥ 5 mm, and into ELI − and ELI + . Chi-square test was used to compare the clinicopathological characteristics. OS and DFS were estimated using Kaplan–Meier method and compared with the log-rank test. Univariable and multivariable Cox proportional hazard models were employed to assess the effect on OS and DFS.

**Results:**

Out of 290 pT3 tumours, 168 (58%) had a depth of tumour invasion < 5 mm and 122 (42%) ≥ 5 mm. The 5-year OS and DFS were 85.2, 68.7 and 60.9%, and 81.4, 73.9 and 60.1% in pT3 < 5 mm, pT3 ≥ 5 mm, and pT4a respectively (*p* = 0.001, *p* = 0.072). Considering ELI − (*n* = 157, 54%) and ELI + (*n* = 133, 46%), the 5-year OS and DFS were 78.9, 76.7, and 60.9%, and 75.5, 81.5, and 60.1% in ELI  − , ELI + and pT4a respectively (*p* = 0.955, *p* = 0.462). At multivariable analysis, the depth of invasion was found to be an independent predictive factor for OS (HR 2.04, 95%CI 1.28–3.24, *p* = 0.003) and DFS (HR 1.98, 95%CI 1.24–3.18, *p* = 0.004), while ELI did not result a prognostic factor for OS nor DFS.

**Conclusion:**

In pT3 colon cancer, depth of tumour invasion ≥ 5 mm is an independent risk factor for OS and DFS, whereas ELI did not result a prognostic factor affecting OS nor DFS.

**Supplementary Information:**

The online version contains supplementary material available at 10.1007/s10147-022-02192-y.

## TNM stage

### Introduction

Colorectal cancer (CRC) is the third most common tumour diagnosed and the third cause of cancer-related deaths in the United States, accounting for 150,000 new estimated cases in 2020 [1]. To date, surgical resection of the tumour with en-bloc removal of the regional lymph nodes is the standard of care for non-metastatic colon cancer.

Pathological stage II represents the most common stage at diagnosis, and shows a variable biological behaviour and outcomes after surgery, with a 5-year overall survival (OS) ranging between 70 and 90% [2–5]. Adjuvant chemotherapy is recommended when high-risk factors are present [6–8]. On the contrary, stage III patients are usually treated with adjuvant chemotherapy to reduce the risk of recurrence [9]. Nevertheless, between 20 and 40% of patients develop recurrence with negative impact on survival [10, 11].

Additional prognostic factors for survival after resection were investigated [12–15]. Whereas in rectal cancer pT3 sub-classification was widely validated [16–18], few studies evaluated additional pathological features for a prognostic subdivision of pT3 colon cancer. Sub-classification of T3 colon cancer based on the depth of infiltration (DOI) beyond the muscularis propria, or peritoneal elastic lamina invasion (ELI) were proposed [19–26]. Yoo et al*.* demonstrated that a subdivision based on the measurement of the maximal DOI is correlated to nodal and distant metastasis and OS [19]. In contrast, Mrak et al. reported that subdivision based on DOI does not provide any additional information about long-term oncologic outcome [20]. Furthermore, supported by the established role of elastic lamina as landmark of visceral pleura invasion in lung cancer staging [27], several studies evaluated peritoneal ELI as a prognostic marker in CRC. Authors reported that pT3 tumours breaching the peritoneal elastic lamina (ELI +) were associated to a worse survival than pT3 tumours not breaching the peritoneal elastic lamina (ELI − ) [23–26]. On the contrary, Grin et al*.* found no significant differences in survival between ELI + and ELI- pT3 tumours in stage II CRC [28].

The aim of our study was to evaluate and compare the clinical significance of pT3 subdivision of intraperitoneal CRC based on the DOI beyond muscularis propria and ELI. To our knowledge, this is the first study that compares these two parameters for a prognostic sub-classification of pT3 colon cancer.

## Methods

### Patients selection

All patients surgically treated for primary CRC from January 1^st^ 2008 to December 31st 2018 were collected from the prospectively maintained colorectal database of the General Surgery 3, University Hospital of Padova. Inclusion criteria were radical resection (R0) for pT3 (study group) and pT4a (control group) colorectal adenocarcinoma. Patients with extraperitoneal rectal adenocarcinoma, who underwent neoadjuvant treatment, with metastatic disease, pT1-pT2-pT4b disease, or histology other than adenocarcinoma were excluded. Patients lost on follow-up were also excluded.

### Clinicopathological and treatment

For each patient data regarding age, sex, body mass index (BMI), ASA score, serum carcinoembryonic antigen (CEA), location (right/left colon), surgical approach, tumour maximal diameter, grading, lymphovascular and perineural invasion, metastatic lymph nodes, and adjuvant treatment were collected. All patients underwent standard oncological surgical resection. Follow-up was performed according to national guidelines [29]. Local recurrence was defined as recurrent disease at the site of the original CRC, whereas distant recurrence was defined as any disease identified outside the primary site.

### Histopathologic analysis

**Fig. 1:**
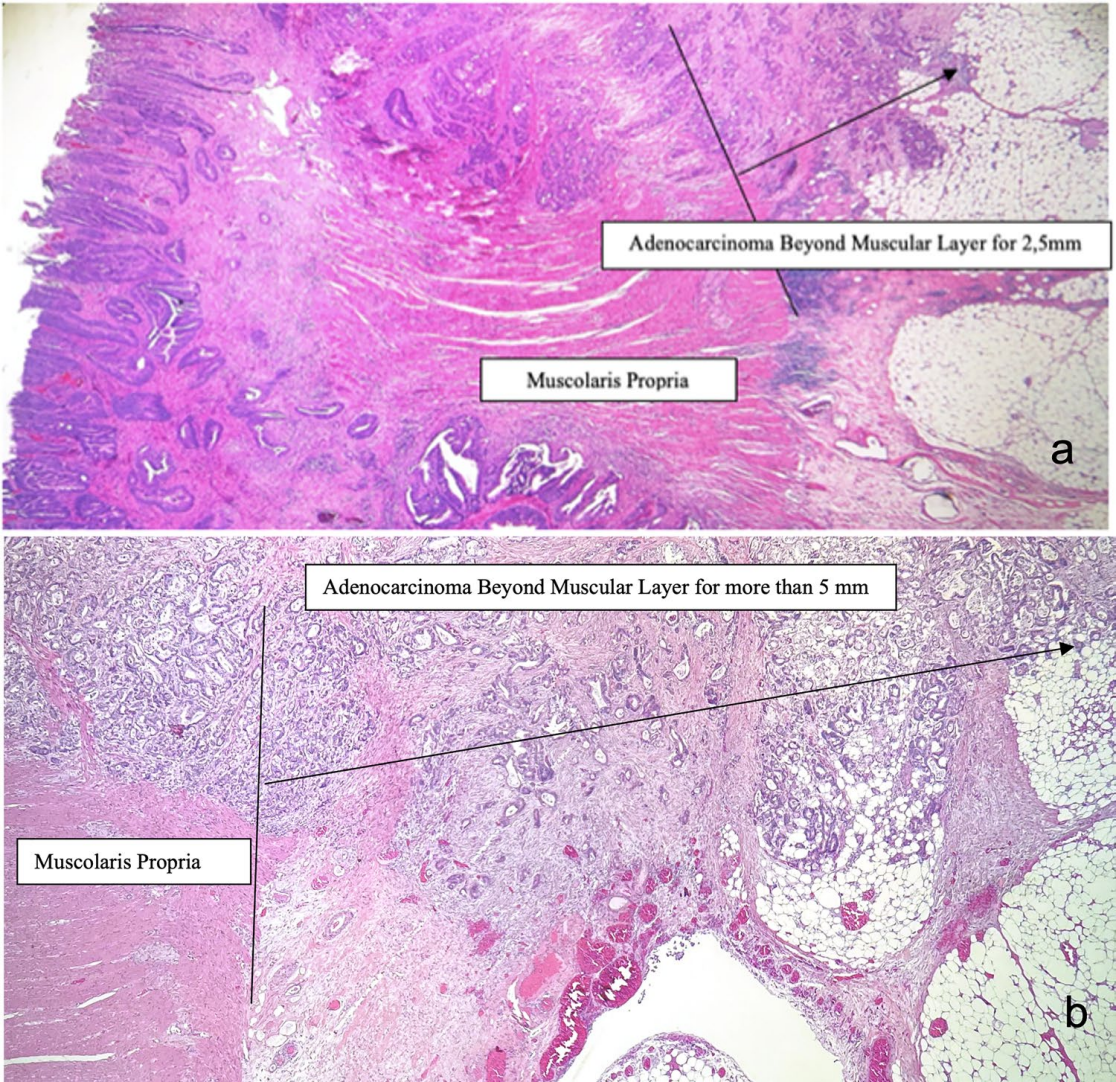
Histology, Hematoxylin and Eosin 25X. Example of depth of infiltration measure. **A** Adenocarcinoma pT3 with depth of tumour invasion < 5 mm. **B**. Adenocarcinoma pT3 with depth of tumour invasion ≥ 5 mm

**Fig. 2:**
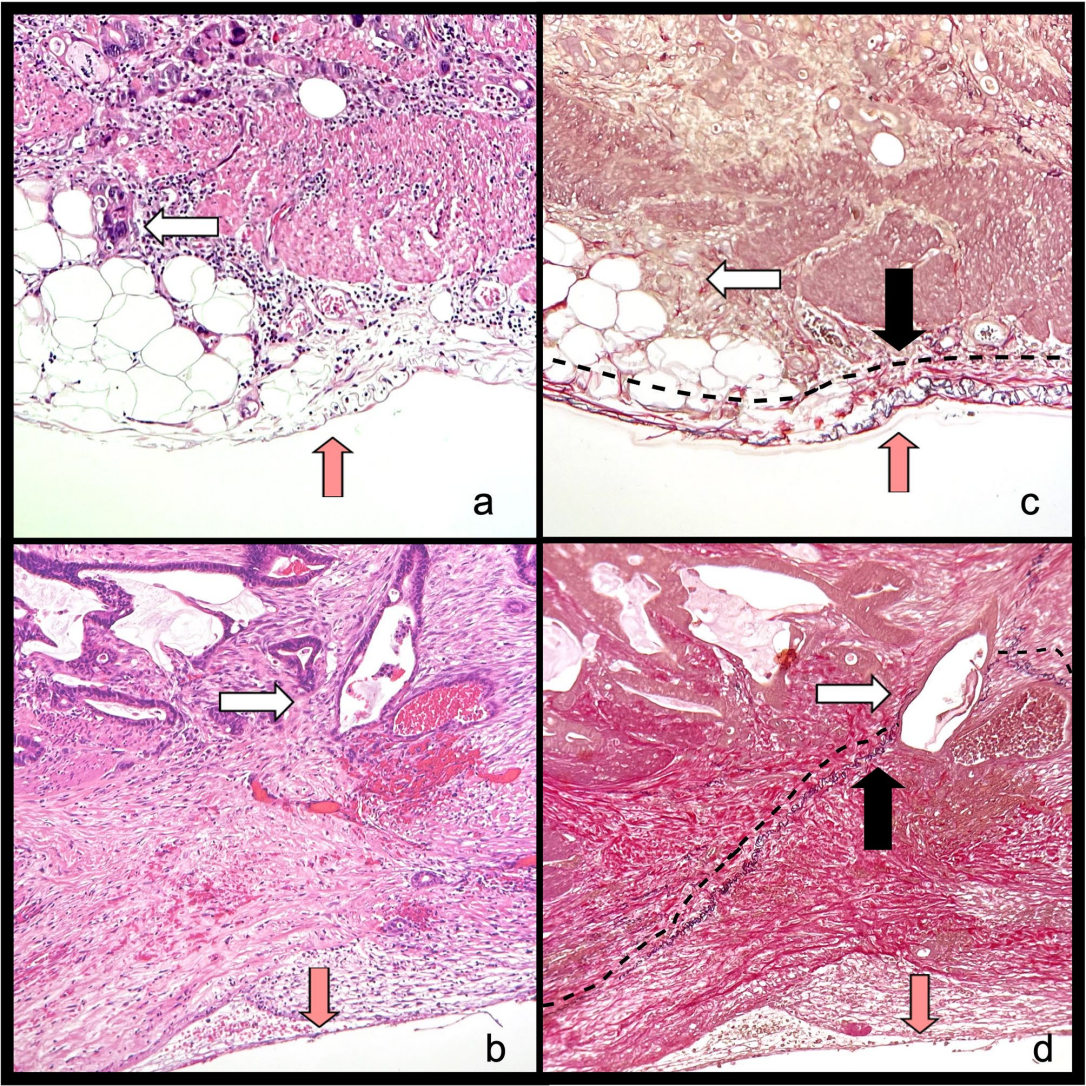
Histology, 100X. Assessment of elastic lamina invasion (ELI). **A** and **B** microphotographs coloured with Hematoxylin and Eosin of adenocarcinoma glands infiltrating fatty tissue near to serosa. White arrows: adenocarcinoma glands; Red arrows: serosa. **C** and **D** microphotographs coloured with Van Gieson&Elastic fibres with elastic lamina coloured in black, **C** adenocarcinoma glands not infiltrating elastic lamina; **D** adenocarcinoma glands infiltrating elastic lamina. White arrows: adenocarcinoma glands; Red arrows: serosa; black arrows and line: elastic lamina

All pathological specimens were staged according to the American Joint Committee on Cancer TNM classification 8th edition [30]. According to our local gross sampling protocol, at least 4 samples from the primary tumour are analysed. All pT3 surgical specimens (*n* = 1160) were jointly reviewed by two gastrointestinal dedicated pathologists, who were blinded to clinical and others pathological data, to assess the sample over four most adequate to determine the DOI. Samples (*n* = 17) with orientation artefacts due to inadequate paraffin embedding were excluded from the selection. DOI was measured in mm from the end of longitudinal muscle layer into the nearby adipose tissue (Fig. 1). The morphometric evaluation of DOI was taken from a dedicated gastrointestinal pathologist with the support of the digital microimaging device Leica DMD108™. The presence of peritoneal ELI was jointly evaluated by two gastrointestinal pathologists, who were blinded to clinical and others pathological data, as landmark for tumour invasion between adipose tissue and serosal layer. Additional elastic stain was performed only in doubtful cases (*n* = 47) (Fig. 2).

### Statistical analysis

Considering the DOI, the study group was divided by a cut-off determined by a Receiver Operating Curve (ROC) for disease-free survival (DFS) (table of sensitivity, specificity and Youden’s J index are shown in Supplementary file, Table 1). Moreover, the study group was divided into two groups, according to the peritoneal ELI, into ELI − and ELI + .

Descriptive statistics was reported as absolute numbers and percentages, and variables distribution in the different subgroups was evaluated using the Chi-squared test. OS and DFS were evaluated using the Kaplan–Meier method and pairwise comparisons were conducted with the log-rank test. P-value associated with the pairwise comparisons underwent Benjamini–Hochberg correction to account for multiplicity of testing. The incidence of recurrence in the subgroups was evaluated using Cumulative Incidence Functions (CIF) in a competing risk framework. Differences in probabilities were evaluated using the Gray’s test. Univariable and multivariable Cox proportional hazard models were employed to assess the effect of the variables of interest on mortality and disease relapse. Results were reported as Hazard Ratio (HR), 95% confidence interval and *p* value. The level for statistical significance was determined at *p* < 0.05. All statistical analyses were performed using R software (version 4.0.3) [31].

## Results

### Clinicopathological characteristics

Clinicopathological characteristics are summarized in Table 1. In the study group (290 patients) there were 164(56.6%) men and 126(43.5%) women, and the median age of was 73(37–95) years. The location of the tumour was right colon in 137(47.2%) and 153(52.8%) left colon. Open surgical approach was performed in 184(63.4%) patients, while a laparoscopic approach in 106(36.6%) patients. Lymph nodes metastasis were retrieved in 121(41.8%) patients. Adjuvant chemotherapy was performed in 143(49.3%) patients. At a median follow-up of 50.5(1–192) months, local and distant recurrence occurred in 9(3.1%) and 51(17.6%) patients respectively. In the control group, 62 pT4a patients was considered for outcomes comparison.

**Table 1:** Patients’ clinicopathological features of Study Group (pT3) and Control Group (pT4a)

	Study group pT3 (*n* = 290)		Control group pT4a (*n* = 62)		Total (*n* = 352)
	*n*	%	*n*	%	
Age					
< 65	105	36.21	17	27.42	122
≥ 65	185	63.79	45	72.58	230
Sex					
Female	126	43.45	32	51.61	158
Male	164	56.55	30	48.39	194
ASA score					
1–2	185	63.79	37	59.68	222
3–4	105	36.21	25	40.32	130
BMI					
< 24	109	37.59	22	35.48	131
≥ 24	181	62.41	40	64.52	221
CEA					
< 5	162	55.86	24	38.71	186
≥ 5	68	23.45	23	37.10	91
NA	60	20.69	15	24.19	75
Location					
Right colon	137	47.24	38	61.29	175
Left colon	153	52.76	24	38.71	177
Laparoscopic					
No	184	63.45	24	38.71	208
Yes	106	36.55	38	61.29	144
Tumour diameter					
< 4 cm	110	38.00	35	56.45	145
≥ 4 cm	180	62.00	27	43.55	207
Grading					
Low	242	83.45	43	69.35	285
High	48	16.55	19	30.65	67
Vascular invasion					
No	105	36.21	12	19.35	117
Yes	185	63.79	50	80.65	235
Lymphatic Invasion					
No	251	86.55	49	79.03	300
Yes	39	13.45	13	20.97	52
Perineural Invasion					
No	162	55.86	22	35.48	184
Yes	128	44.14	40	64.52	168
Positive lymph nodes					
No	169	58.28	24	38.71	193
Yes	121	41.72	38	61.29	159
Adjuvant CT					
No	147	50.69	21	33.87	168
Yes	143	49.31	41	66.13	184
Local recurrence					
No	281	96.90	61	98.39	342
Yes	9	3.10	1	1.61	10
Distant recurrence					
No	239	82.41	41	66.13	280
Yes	51	17.59	21	33.87	72

### Depth of tumour infiltration

**Fig. 3:**
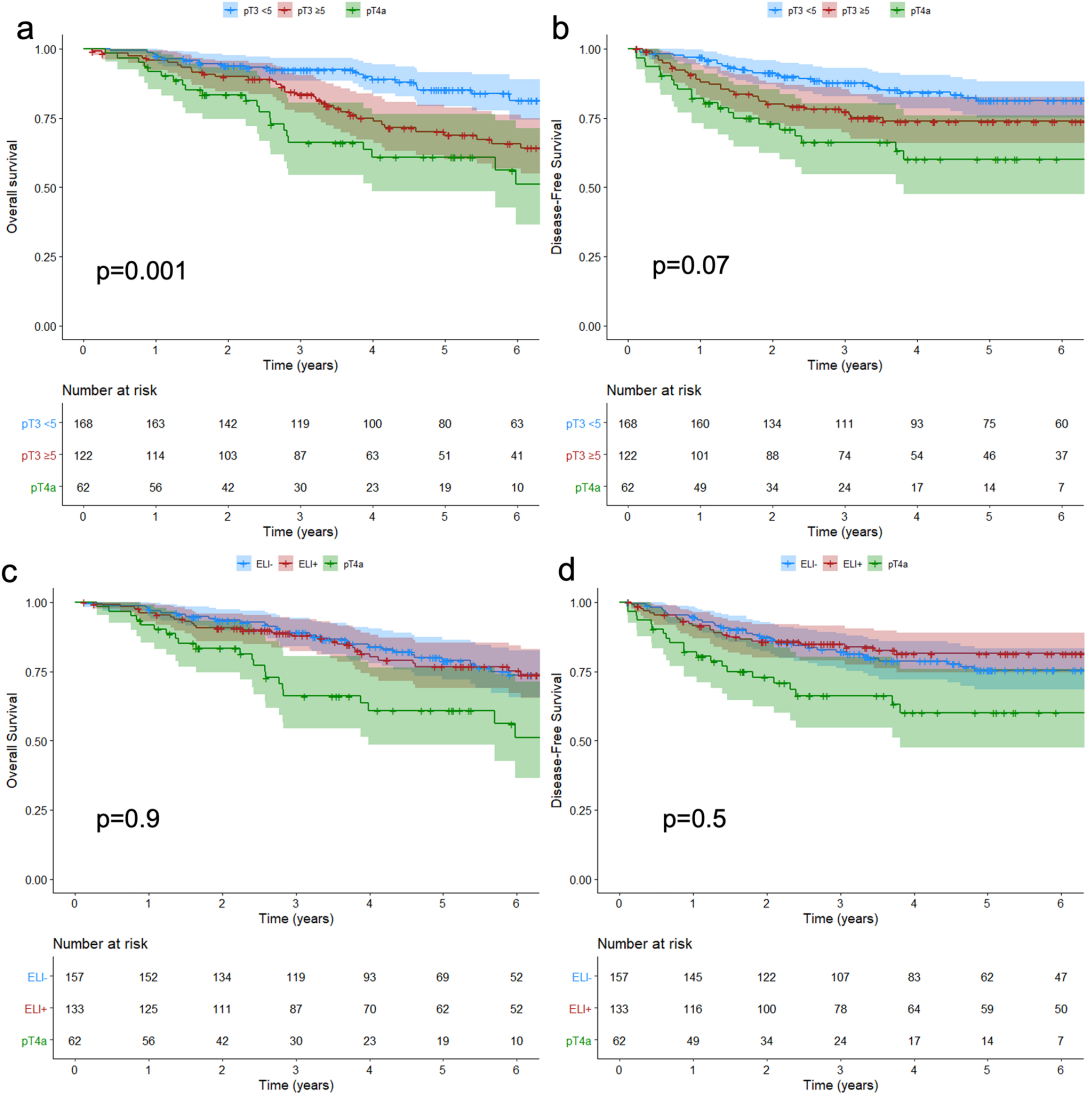
Kaplan–Meier Survival Estimates. **A** OS in patients with pT3 < 5 mm and pT3 ≥ 5 mm and pT4a. **B** DFS in patients with pT3 < 5 mm and pT3 ≥ 5 mm and pT4a. **C **OS in patients with ELI -, ELI + and pT4a. **D** DFS in patients with ELI −, ELI + and pT4a

The median value of maximum DOI of pT3 tumour infiltration was 4.0 (0.2–25) mm. The ROC curve was obtained by correlating the maximum DOI with DFS, identifying a depth of 5 mm as the best cut-off, with an area under the curve (AUC) of 0.5916 (Supplementary file, Table 1 and Fig. 1). According to this cut-off, the study cohort was divided in pT3 < 5 mm and pT3 ≥ 5 mm (Table 2). The estimated 5-year OS were 85.2, 68.8, and 60.9% in pT3 < 5 mm, pT3 > 5 mm, and pT4a respectively (log-rank test pT3 < 5 mm Vs pT3 ≥ 5 mm: *p* = 0.001); the estimated 5-year DFS were 81.4, 73.9, and 60.1% in pT3 < 5 mm, pT3 ≥ 5 mm, and pT4a respectively (log-rank test pT3 < 5 mm Vs pT3 > 5 mm: *p* = 0.07) (Fig. 3a, Fig. 3b).

**Table 2:** Patients’ clinical features and correlation to the depth of tumour invasion (pT3 < 5 mm and pT3 ≥ 5 mm)

	pT3 < 5 mm (*n* = 168)		pT3 ≥ 5 mm (*n* = 122)		*p* value
	*n*	%	*n*	%	
Age					
< 65	61	36.30	35	28.70	0.17
≥ 65	107	63.70	87	71.30	
Sex					
F	67	39.90	59	48.40	0.15
M	101	60.10	63	51.60	
ASA score					
1–2	106	63.10	79	64.80	0.77
3–4	62	36.90	43	35.20	
BMI					
< 30	144	85.70	101	82.80	0.49
≥ 30	24	14.30	21	17.20	
CEA					
< 5	106	63.10	56	45.90	0.009
≥ 5	32	36.90	36	29.50	
NA	30	18.00	30	24.60	
Location					
Right colon	80	47.60	61	50.00	0.68
Left colon	88	52.40	61	50.00	
Laparoscopic					
No	95	56.50	89	73.00	0.004
Yes	73	43.50	33	27.00	
Tumour diameter					
< 4 cm	74	66.70	63	51.60	0.012
≥ 4 cm	94	33.30	59	48.40	
Grade					
Low	145	86.00	97	80.00	0.124
High	23	14.00	25	20.00	
Vascular invasion					
No	66	39.30	39	32.00	0.20
Yes	102	60.70	83	68.00	
Lymphatic invasion					
No	146	86.90	105	86.00	0.83
Yes	22	13.10	17	14.00	
Perineural invasion					
No	93	55.35	69	56.55	0.83
Yes	75	44.65	53	43.45	
Positive lymph nodes					
No	112	66.70	63	51.60	0.01
Yes	56	33.30	59	48.40	
Adjuvant CT					
No	85	50.60	62	50.80	0.97
Yes	83	49.40	60	49.20

### Elastic lamina invasion

pT3 ELI − included 157 (54.1%) tumours and 133 (45.9%) pT3 ELI + (Table 3). The 5-years OS and DFS were 78.9, 76.7%, and 60.9%, and 75.5, 81.5, and 60.1% in ELI −, ELI + and pT4a respectively (log-rank test ELI + Vs ELI −: *p* = 0.9 for OS and *p* = 0.5 for DFS) (Fig. 3c, Fig. 3d).

**Table 3:** Patients’ clinical features and correlation to peritoneal elastic lamina invasion

	ELI – (*n* = 157)		ELI + (*n* = 133)		*p* value
	*n*	%	*n*	%	
Age					
< 65	48	30.60	48	36.10	0.32
≥ 65	109	69.40	85	63.90	
Sex					
Female	102	65.00	62	46.60	0.002
Male	55	35.00	71	53.40	
ASA score					
1–2	100	63.70	85	63.90	0.97
3–4	57	36.30	48	36.10	
BMI					
< 30	135	86.00	110	82.70	0.44
≥ 30	22	14.00	23	17.30	
CEA					
< 5	94	59.90	68	51.10	0.08
≥ 5	31	19.70	37	27.80	
NA	32	20.40	28	21.10	
Location					
Right colon	71	45.20	70	52.60	0.20
Left colon	86	54.80	63	47.40	
Laparoscopic					
No	99	63.10	85	63.90	0.88
Yes	58	36.90	48	36.10	
Tumour diameter					
< 4 cm	63	40.10	47	35.30	0.40
≥ 4 cm	94	59.90	86	64.70	
Grading					
Low	140	89.00	102	77.00	0.004
High	17	11.00	13	23.00	
Vascular invasion					
No	60	38.20	45	33.80	0.43
Yes	97	61.80	88	66.20	
Lymphatic invasion					
No	137	87.30	114	85.70	0.70
Yes	20	12.70	19	14.30	
Perineural invasion					
No	95	60.50	67	50.40	0.08
Yes	62	39.50	66	49.60	
Positive lymph nodes					
No	106	67.50	69	51.90	0.007
Yes	51	32.50	64	48.10	
Adjuvant CT					
No	82	52.20	65	48.90	0.56
Yes	75	47.80	68	51.10	

### Univariable and multivariable analysis

At univariable analysis, factors significantly associated with a decreased OS were age, ASA score, lymphatic invasion, lymph nodes metastasis, and DOI (pT3 ≥ 5 mm) (Table 4). Adjuvant chemotherapy resulted as protective factor (HR 0.54, 95% CI 0.34–0.86, *p* = 0.096). At multivariable analysis, independent risk factors were age (HR 2.93, 95% CI 1.44–5.93, *p* = 0.003), ASA score (HR 2.59, 95% CI 1.59–4.20, *p* < 0.001), lymph nodes metastasis (HR 1.78, 95% CI 1.11–2.85, *p* = 0.015) and DOI (HR 2.04, 95% CI 1.28–3.24, *p* = 0.003).

**Table 4:** Univariable and multivariable analysis for overall survival (OS) and disease-free survival (DFS)

	Univariate analysis for OS			Multivariate analysis for OS			Univariate analysis for DFS			Multivariate analysis for DFS		
	HR	95% CI	*p *value	HR	95% CI	*p* value	HR	95% CI	*p* value	HR	95% CI	*p* value
Age (≥ 65)	4.34	(2.22–8.46)	< 0.001	2.93	(1.44–5.93)	0.003	1.51	(0.90–2.54)	0.113			
Sex (male)	1.05	(0.67–1.66)	0.816				1.64	(1.00–2.67)	0.047	1.56	(0.95–2.56)	0.076
ASA > 2	3.50	(2.21–5.56)	< 0.001	2.59	(1.59–4.20)	< 0.001	1.43	(0.89–2.29)	0.133			
BMI (≥ 30)	0.79	(0.38–1.65)	0.536				1.08	(0.57–2.06)	0.794			
CEA ≥ 5 ng/ml	1.31	(0.74–2.30)	0.339				1.25	(0.71–2.20)	0.433			
Left colon site	0.65	(0.41–1.03)	0.071				1.30	(0.81–2.07)	0.268			
Laparoscopy	0.58	(0.34–1.00)	0.052	0.77	(0.42–1.39)	0.394	1.36	(0.85–2.18)	0.190			
Tumour ≥ 4 cm	0.69	(0.44–1.09)	0.120				0.72	(0.45–1.15)	0.171			
High grade	1.03	(0.57–1.88)	0.908				0.73	(0.33–1.62)	0.451			
VI	1.19	(0.75–1.89)	0.458				1.42	(0.87–2.31)	0.159			
LI	1.94	(1.01–3.74)	0.046	1.79	(0.91–3.53)	0.090	2.11	(1.17–3.81)	0.013	1.70	(0.93–3.10)	0.081
PNI	1.39	(0.87–2.20)	0.160				2.19	(1.37–3.48)	0.001	1.69	(1.03–2.76)	0.034
N +	1.70	(1.08–2.67)	0.021	1.78	(1.11–2.85)	0.015	2.76	(1.73–4.42)	< 0.001	2.14	(1.20–3.80)	0.009
LN ≥ 12	0.71	(0.44–1.16)	0.178				0.50	(0.29–0.87)	0.016	0.41	(0.22–0.74)	0.004
Adjuvant CT	0.54	(0.34–0.86)	0.010	0.62	(0.36–1.08)	0.096	1.23	(0.77–1.96)	0.375			
pT3 ≥ 5 mm	2.16	(1.36–3.42)	0.001	2.04	(1.28–3.24)	0.003	1.86	(1.17–2.96)	0.008	1.98	(1.24–3.18)	0.004
ELI	0.98	(0.62–1.55)	0.963				0.93	(0.93–1.22)	0.776			

*BMI* body mass index, *CEA* carcinoembryonic antigen, *VI* vascular invasion, *LI* lymphatic invasion, *PNI* perineural invasion, *N* + lymph nodes metastasis, *LN* examined lymph nodes, *Adjuvant CT* Adjuvant chemotherapy, *ELI* elastic lamina invasion

Factors significantly associated with a decreased DFS were sex, lymphatic invasion, perineural invasion, lymph nodes metastasis, and DOI (Table 4). Examined lymph nodes ≥ 12 resulted as protective factor (HR 0.50, 95%CI 0.29–0.87, *p* = 0.016). At multivariable analysis, independent risk factors for DFS were perineural invasion (HR 1.69, 95%CI 1.03–2.76, *p* = 0.034), lymph nodes metastasis (HR 2.14, 95%CI 1.20–3.80, *p* = 0.009), and DOI (HR 1.98, 95%CI 1.24–3.18, *p* = 0.004). Examined lymph nodes > 12 was confirmed as protective factor (HR 0.41, 95%CI 0.22–0.74, *p* = 0.004). ELI did not result associated at univariable analysis with OS (HR 0.98, 95%CI 0.62–1.55, *p* = 0.963) nor DFS (HR 0.93, 95%CI 0.93–1.22, *p* = 0.776). Association between DOI and ELI, and lymph nodes metastasis was also examined using a univariable logistic regression approach. Both pT3 ≥ 5 mm and ELI + were factors related to lymph nodes metastasis (OR 1.87, 95%CI 1.61–3.02, *p* = 0.010, OR 1.928, 95%CI 1.197–3.105, *p* = 0.007 respectively).

### Sub-analysis according to N status

Considering only N0 patients (169 patients), the 5-year OS was respectively 86.8, and 85.6% in pT3 < 5 mm and pT3 ≥ 5 mm, and the 5-year DFS were 83.4, and 88.6% (log-rank test *p* = 0.72 and *p* = 0.52) (Supplementary file, Fig. 2). Estimated 5-year recurrence rate was 15.9 and 10.8% in pT3 < 5 mm, pT3 ≥ 5 mm respectively (Grey’s test *p* = 0.48) (Supplementary file, Fig. 3). At univariable analysis, DOI did not result associated with an impaired OS nor DFS (HR 1.130, 95%CI 0.582–2.191, *p* = 0.719; HR 0.731, 95%CI 0.281–1.903, *p* = 0.521) (Supplementary file, Table 2 and Table 3).

**Fig. 4:**
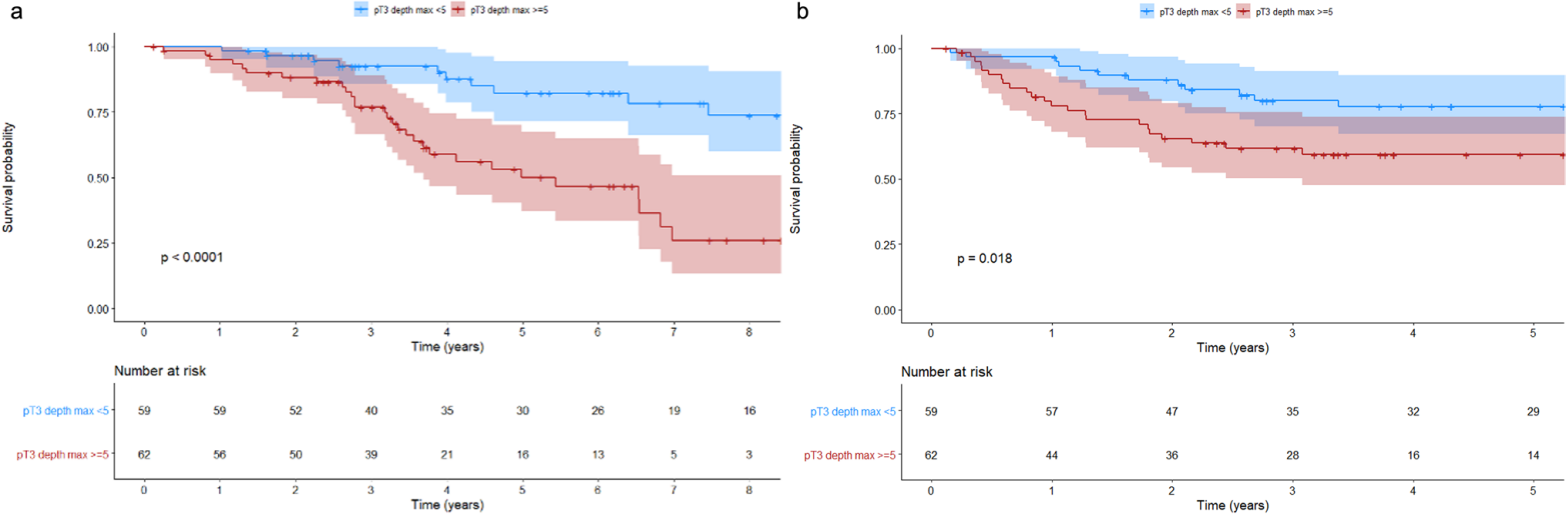
Kaplan–Meier Survival Estimates for **a** Overall survival (OS) and **b** Disease-free survival (DFS) in pN + patients. Patients with pT3 < 5 mm N + (depth of invasion < 5 mm) and pT3 ≥ 5 mm N + (depth of invasion ≥ 5 mm)

Considering only N + patients (121 patients), the 5-year OS was respectively 82.1 and 50% in pT3 < 5 mm and pT3 ≥ 5 mm, and the 5-year DFS was 77.7 and 59.3% (log-rank test *p* < 0001 and *p* = 0.02) (Fig. 4). Estimated 5-year recurrence rate was 22.0 and 39.4%, 41.4 and 21.9% in pT3 < 5 mm, pT3 ≥ 5 mm, ELI − and ELI + respectively (Grey’s test *p* = 0.023 and *p* = 0.069 respectively) (Supplementary file, Fig. 5). At multivariable analysis, DOI pT3 ≥ 5 mm resulted associated with an impaired OS and DFS (HR 3.26, 95% CI 1.564–6.77, *p* = 0.002) (HR 2.309, 95% CI 1.147–4.645, *p* = 0.019), while at univariable analysis ELI did not result a predictive factor for OS and DFS (HR 0.720, 95% CI 0.387–1.340, *p* = 0.521, HR 0.547, 95% CI 0.278–1.076, *p* = 0.081) (Table 5).

**Table 5:** Univariable and multivariable analysis for (OS) and Disease-Free Survival (DFS) in pT3N + patients

	Univariate analysis for OS			Multivariate analysis for OS			Univariate analysis for DFS			Multivariate analysis for DFS		
	HR	95% CI	*p* value	HR	95% CI	*p* value	HR	95% CI	*p* value	HR	95% CI	*p* value
Age ≥ 65	4.82	(2.02–11.55)	< 0.001	3.94	(1.63–9.56)	0.002	1.55	(0.76–3.16)	0.232			
Sex (Male)	0.66	(0.36–1.23)	0.192				0.98	(0.50–1.91)	0.944			
ASA > 2	2.46	(1.32–4.61)	0.005	–	–	–	1.21	(0.61–2.37)	0.588			
BMI ≥ 30	1.72	(0.86–3.47)	0.126				1.60	(0.77–3.33)	0.210			
CEA ≥ 5 ng/ml	2.51	(1.35–4.69)	0.004	2.55	(1.32–4.92)	0.005	1.42	(0.72–2.79)	0.311			
Left colon site	0.71	(0.38–1.32)	0.281				1.23	(0.62–2.45)	0.548			
Laparoscopy	0.42	(0.19–0.91)	0.028	–	–	–	1.18	(0.60–2.33)	0.625			
Tumour ≥ 4 cm	0.88	(0.47–1.66)	0.693				0.73	(0.38–1.42)	0.353			
High grade	1.08	(0.50–2.35)	0.840				0.61	(0.24–1.57)	0.305			
Adjuvant CT	0.38	(0.20–0.72)	0.003	–	–	–	0.70	(0.34–1.43)	0.327			
VI	0.94	(0.44–1.97)	0.859				1.35	(0.53–3.49)	0.531			
PNI	1.16	(0.61–2.20)	0.646				2.31	(1.05–5.09)	0.038	2.35	(1.07–5.17)	0.034
LI	2.22	(1.06–4.63)	0.034	3.31	(1.50–7.31)	0.003	1.75	(0.84–3.65)	0.138			
LN ≥ 12	0.80	(0.38–1.68)	0.556				0.58	(0.27–1.25)	0.164			
pT3 ≥ 5 mm	3.97	(1.94–8.11)	< 0.001	3.26	(1.56–6.78)	0.002	2.27	(1.13–4.58)	0.021	2.31	(1.15–4.65)	0.019
ELI	0.72	(0.39–1.34)	0.300				0.55	(0.28–1.08)	0.081			

*BMI* body mass index, *CEA*   carcinoembryonic antigen, *Adjuvant CT*   Adjuvant chemotherapy, *VI*   vascular invasion, *LI*   lymphatic invasion, *PNI* = perineural invasion, *N* +   lymph nodes metastasis, *LN*   examined lymph nodes, *ELI*  elastic lamina invasion

## Discussion

Assessment of additional pathological features might allow an accurate risk stratification to identify high-risk stage II CRC patients who benefit from adjuvant therapy. A pT3 sub-classification based on DOI was proposed by several authors, while others strongly suggested a sub-classification based on peritoneal ELI.

We used a subdivision of pT3 using a cut-off, determined by using a ROC for DFS, of 5 mm DOI beyond the muscular layer, which resulted to affect survival. A DOI > 15 mm was proposed by Merkel et al*.* as a major risk factors for stage II colon carcinoma [32], while various cut-offs between 2 and 10 mm were demonstrated for the subdivision of T3 CRC [16, 33]. Yoo et al., similarly to Pollheimer et al., divided pT3 patients using 4 different cut-offs (< 1 mm; 1–5 mm; 5–15 mm; > 15 mm), reporting 5 mm cut-off as the strongest prognostic factor in both their analysis [19, 34]. Recently, Nomura et al*.* reported 148 pT3 CRC subdivided into T3a (< 1 mm); T3b (1–5 mm); T3c (> 5 mm) and confirmed a 5 mm as optimal cut-off predictor for recurrence [21].

At univariable and multivariable analysis pT3 ≥ 5 mm category resulted as independent prognostic factor both for OS and DFS. Similarly, Bori et al*.* divided 593 CRC by a 5 mm cut-off, reporting that pT3 < 5 mm is associated with an improved long-term outcomes in terms of nodal involvement and distant metastasis [35]. Furthermore, similar results were reported by several authors [19, 21, 36], in particular Akagi et al*.* reported that pT3 ≥ 5 mm was the strongest independent risk factor for recurrence on 202 cases of stage II colon cancer, and proposed adjuvant chemotherapy as indicated for these patients [37]. Based on this rationale, our sub-analysis on pT3N0 and pT3N + aimed to determine if the DOI ≥ 5 mm may be a recommendation for adjuvant treatment. In this setting, the DOI failed to result as predictive factors for survival in pT3N0 patients, although it was confirmed as a strong independent predictor of OS and DFS in pT3N + patients (HR 3.26, 95%CI 1.56–6.78, *p* = 0.002, and HR 2.31 95%CI 1.15–4.65, *p* = 0.019 respectively). In this setting, DOI confirmed to be an index of advanced disease in pT3N + patients, as far as Kaplan–Meier survival analysis showed that in N + group pT3 ≥ 5 mm patients had a survival even worse than pT4 control group (5-year OS 50.0 Vs 60.9%, 5-year DFS 59.3 Vs 60.1% respectively). For this reason, we also strongly suggest to consider DOI as a high-risk factor, that may help in case of doubt for adjuvant treatment, even in pT3N0 patients.

Alternatively to the measurement of DOI, Shinto et al. used the peritoneal ELI as a cut-off, finding an increased recurrence rate and decrease of survival for tumours with ELI [24]. Kojima et al. reported that ELI had a strong impact on survival and may be useful as a pathologic diagnostic tool to predict high-risk colon cancer [23]. In contrast, Grin et al*.* observed no significant differences in DFS between 186 patients with pT3 ELI- and ELI + stage II CRC [28]. In particular, they underlined the limitation of ELI as predictive factor, since in 18.3% of the cases the elastic lamina was not identifiable elastic lamina, mostly in right-sided tumours, despite repeated staining and assessment of multiple blocks. Even when identifiable, the ELI assessment was often challenging, because of severe distortion or destruction caused by a fibroinflammatory reaction close to the tumour [22]. To note, in our analysis ELI resulted recognizable in every patient during pathological revision and elastic stain were performed only in doubtful cases. To overcome this limitation, Liang et al. and Nakanishi et al. sustained the routine use of elastic stain as a useful and inexpensive method to demonstrate peritoneal ELI by tumour that should be considered for routine use in all CRC [26, 38]. Lu et al., proposed ELI assessed with elastic stain as prognostic factors for stage II colon cancer, and might be an indication to postoperative adjuvant chemotherapy [39]. Recently, a meta-analysis including six studies, recommended the sub-categorization of pT3 CRC by ELI for better prognostic assessment and treatment strategy of patients with CRC [40]. Our analysis failed to confirm any significant impact of ELI on oncological outcome, and ELI as predictive factor at univariable analysis. To note, lymph nodes metastasis and adjuvant treatment have a main role in long-term outcome, and the prognostic role of ELI may have been mitigated by the high rate of Stage III who underwent adjuvant treatment (70% in ELI + in pT3N + patients). As reported by Yokota et al., even if ELI resulted as independent risk factor for RFS and OS, RFS resulted almost identical when comparing pT3N + /ELI + to pT3N + /ELI- in patients with no adjuvant treatment [25].

Along with DOI and ELI, the circumferential resection margin (CRM) was investigated in colon cancer also, whereas its prognostic role in rectal cancer is well known. NCDB registry showed that a positive CRM were common in pT4 (26–32% of patients), whereas in pT2 and pT3 colon cancer is less common (6 and 11% of patients respectively) [41]. A positive CRM should be an indication for adjuvant treatment in stage II colon cancer, however in only 9% of these patients were reported [42]. Nevertheless, the histopathological parameters that we considered are more common in T3 colon cancer patients, representing in both cases more than 40% of the patients. Furthermore, beside pathological analysis in the last years circulating tumour DNA (ctDNA) is assuming an increasing role in the prognosis of colon cancer. Our group previously reported that an increased level of ctDNA was associated to a poor prognosis [43], whereas an Australian multicenter RCT reported ctDNA as independent predictors of recurrence-free survival [44]. Unfortunately, even considering these promising data, the ctDNA is not commonly tested in the normal follow-up of colon cancer, whereas the evaluation of DOI and ELI are an unexpensive extension of normal pathological examinations.

There are some limitations in this study. First, this is a single-institution retrospective study. Even if we considered the limitation of a single institution study, in our study group almost 300 colon cancer were re-evaluated by dedicated colorectal pathologists. Second, the patients included in the study covered a wide timespan of 10 years, during which several changes occurred in staging modalities, preoperative treatment, anaesthesiological, pathological, and surgical techniques, follow-up and adjuvant treatment. All these changes may have a potential impact on OS and DFS. Lastly, we did not identify other confounding factors, such as high-risk features in Stage II or Stage III not treated with adjuvant therapy. In our cohort high-risk stage II and stage III patients routinely underwent adjuvant treatment. Patients in these stages who did not receive any adjuvant treatment might have been unfit for further treatment, and this may have an adverse impact on survival. For these reasons, the results of a further subgroup analysis by considering these confounding factors may not be reliable also.

## Conclusion

In our study, the DOI beyond the muscular layer of colonic wall, using a cut-off of 5 mm, is an independent risk factor both for OS and DFS, and may be considered as high-risk feature in pT3 colon cancer. ELI was not resulted to be a prognostic factor affecting OS and DFS. To our knowledge, this is the first study that compares these 2 parameters for a prognostic sub-classification of pT3 colon cancer.

## Supplementary Information

Below is the link to the electronic supplementary material.

Supplementary file1 (DOCX 186 KB)
