# Supplementary material for: Prognostic significance of additional histologic features for subclassification of pathological T3 colon cancer
Source: Int J Clin Oncol. 2022 Jun 18;27(9):1428–38. doi: 10.1007/s10147-022-02192-y (PMC9393148; doi:10.1007/s10147-022-02192-y)
Supplement: Supplementary file 1 — Supplementary file1 (DOCX 186 KB) [file 10147_2022_2192_MOESM1_ESM.docx]

**Supplementary File**

**Table 1. Sensitivity, specificity and Youden's J**

| Interval | Sensitivity | Specificity | J-index |
| --- | --- | --- | --- |
| (>=0.2) | 100.00% | 0.00% | - |
| (>=0.5) | 95.52% | 5.00% | 0.01 |
| (>=0.8) | 85.07% | 18.00% | 0.03 |
| (>=1.5) | 82.09% | 19.50% | 0.02 |
| (>=2) | 79.10% | 25.50% | 0.05 |
| (>=2.5) | 70.15% | 32.50% | 0.03 |
| (>=3) | 67.16% | 34.00% | 0.01 |
| (>=3.5) | 65.67% | 45.50% | 0.11 |
| (>=4) | 65.67% | 49.50% | 0.15 |
| (>=4.5) | 59.70% | 59.00% | 0.19 |
| (>=5) | **53.73%** | **65.00%** | **0.19** |
| (>=5.5) | 47.76% | 68.50% | 0.16 |
| (>=6) | 43.28% | 72.00% | 0.15 |
| (>=6.5) | 38.81% | 77.50% | 0.16 |
| (>=7) | 35.82% | 80.00% | 0.16 |
| (>=7.5) | 32.84% | 83.50% | 0.16 |
| (>=8) | 31.34% | 86.50% | 0.18 |
| (>=8.5) | 25.37% | 88.50% | 0.14 |
| (>=9) | 23.88% | 89.00% | 0.13 |
| (>=10) | 22.39% | 90.50% | 0.13 |
| (>=11) | 20.90% | 91.00% | 0.12 |
| (>=11.5) | 14.93% | 92.50% | 0.07 |
| (>=12) | 13.43% | 92.50% | 0.06 |
| (>=13) | 8.96% | 93.50% | 0.02 |
| (>=13.5) | 7.46% | 94.00% | 0.01 |
| (>=14) | 7.46% | 94.50% | 0.02 |

**Table 2. Univariable and multivariable analysis for Overall Survival (OS) in pT3N0**

|  | **Univariable for OS** | | | | **Multivariable for OS** | | | |
| --- | --- | --- | --- | --- | --- | --- | --- | --- |
|  | **HR** | **Lower IC 95%** | **Upper IC 95%** | **P-value** | **HR** | **Lower IC 95%** | **Upper IC 95%** | **P-value** |
| Age | 4.588 | 1.618 | 13.010 | 0.004 | 2.139 | 0.667 | 6.859 | 0.201 |
| Sex | 1.719 | 0.868 | 3.404 | 0.120 |  |  |  |  |
| ASA score ≥ 3 | 4.891 | 2.441 | 9.798 | <0.001 | 3.235 | 1.457 | 7.186 | 0.004 |
| BMI ≥24 | 0.942 | 0.479 | 1.853 | 0.862 |  |  |  |  |
| CEA ≥5 | 1.234 | 0.637 | 2.391 | 0.533 |  |  |  |  |
| Left colon site | 0.564 | 0.283 | 1.124 | 0.103 |  |  |  |  |
| Laparoscopy | 0.795 | 0.371 | 1.703 | 0.555 |  |  |  |  |
| Tumor ≥4cm | 0.711 | 0.368 | 1.374 | 0.310 |  |  |  |  |
| High Grade | 0.942 | 0.365 | 2.432 | 0.902 |  |  |  |  |
| Adjuvant CT | 0.330 | 0.144 | 0.756 | 0.009 | 0.684 | 0.275 | 1.701 | 0.414 |
| VI | 0.973 | 0.500 | 1.894 | 0.936 |  |  |  |  |
| LI | 1.026 | 0.465 | 2.266 | 0.950 |  |  |  |  |
| PNI | 0.605 | 0.082 | 4.479 | 0.623 |  |  |  |  |
| LN ≥12 | 0.576 | 0.297 | 1.118 | 0.103 |  |  |  |  |
| pT3≥5mm | 1.130 | 0.582 | 2.191 | 0.719 |  |  |  |  |
| ELI | 1.097 | 0.567 | 2.124 | 0.784 |  |  |  |  |

**Table 3. Univariable analysis for Disease-free survival. (DFS) in pT3N0**

|  | **HR** | **Lower IC 95%** | **Upper IC 95%** | **P-value** |
| --- | --- | --- | --- | --- |
| Age | 2.698 | 0.790 | 9.210 | 0.113 |
| Sex | 1.983 | 0.762 | 5.162 | 0.161 |
| ASA score ≥ 3 | 2.373 | 0.986 | 5.712 | 0.054 |
| BMI ≥24 | 1.434 | 0.545 | 3.774 | 0.465 |
| CEA ≥5 | 1.425 | 0.590 | 3.443 | 0.431 |
| Left colon site | 1.424 | 0.573 | 3.542 | 0.447 |
| Laparoscopy | 1.758 | 0.726 | 4.256 | 0.211 |
| Tumor ≥4cm | 0.472 | 0.195 | 1.141 | 0.096 |
| High Grade | 0.670 | 0.155 | 2.891 | 0.592 |
| Adjuvant CT | 0.939 | 0.374 | 2.357 | 0.894 |
| VI | 0.510 | 0.196 | 1.330 | 0.169 |
| LI | 1.278 | 0.490 | 3.333 | 0.616 |
| PNI | 1.634 | 0.376 | 7.092 | 0.512 |
| LN ≥12 | 0.288 | 0.119 | 0.694 | 0.006 |
| pT3≥5mm | 0.731 | 0.281 | 1.903 | 0.521 |
| ELI | 1.083 | 0.443 | 2.650 | 0.861 |

**Figure 1. Receiver Operating Curve (ROC) of the maximum depth of tumor invasion compared to Disease-Free Survival.** Area Under Curve (AUC) 0.5916.


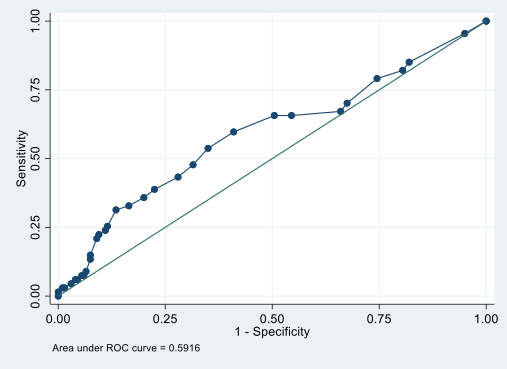


**Figure 2a. Kaplan Meier Survival Estimates in pN0 patients for OS.**


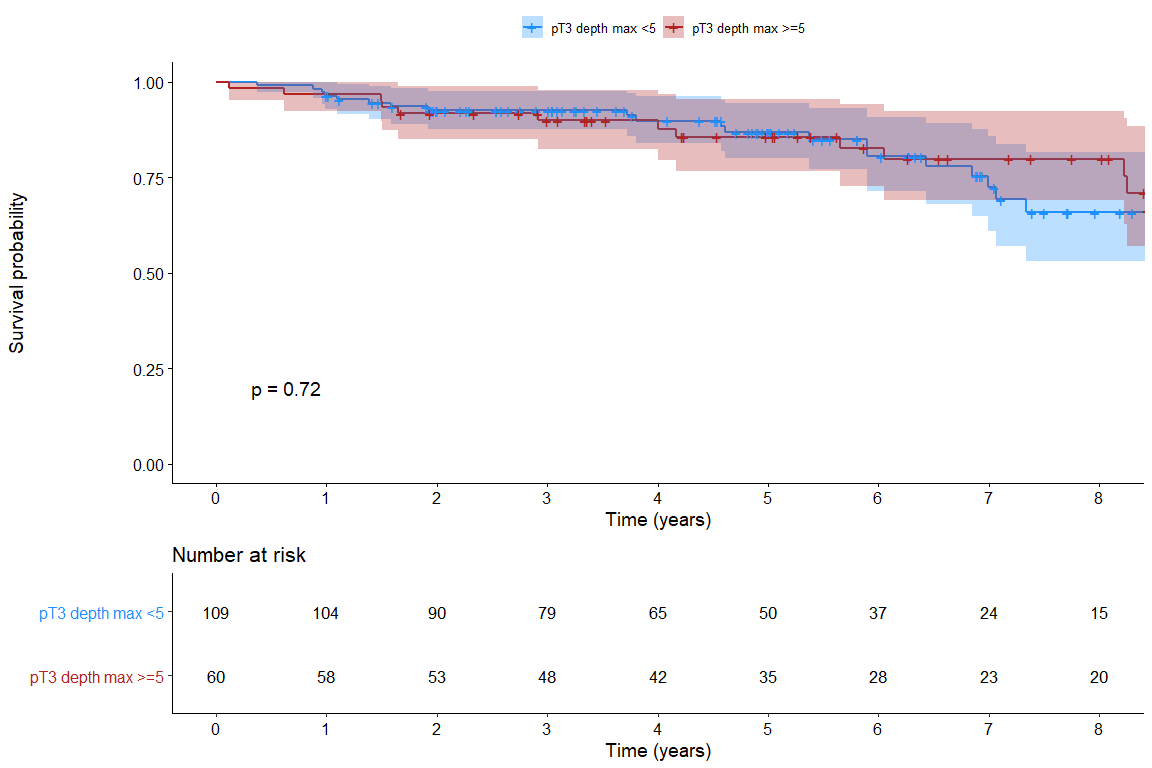


**Figure 2b. Kaplan Meier Survival Estimates in pN0 patients for DFS.**


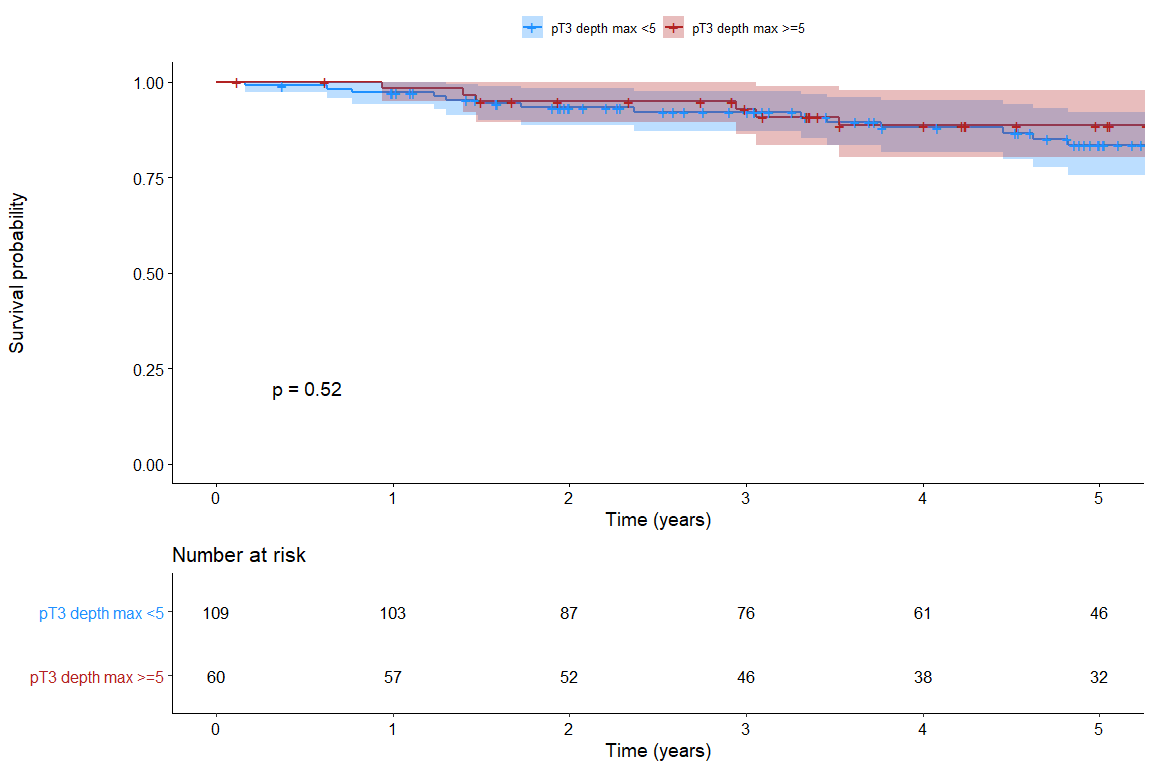


**Figure 3a. Estimated Recurrence Risk (Cumulative incidence function) in N0 patients, in pT3>5mm and pT3<5mm**


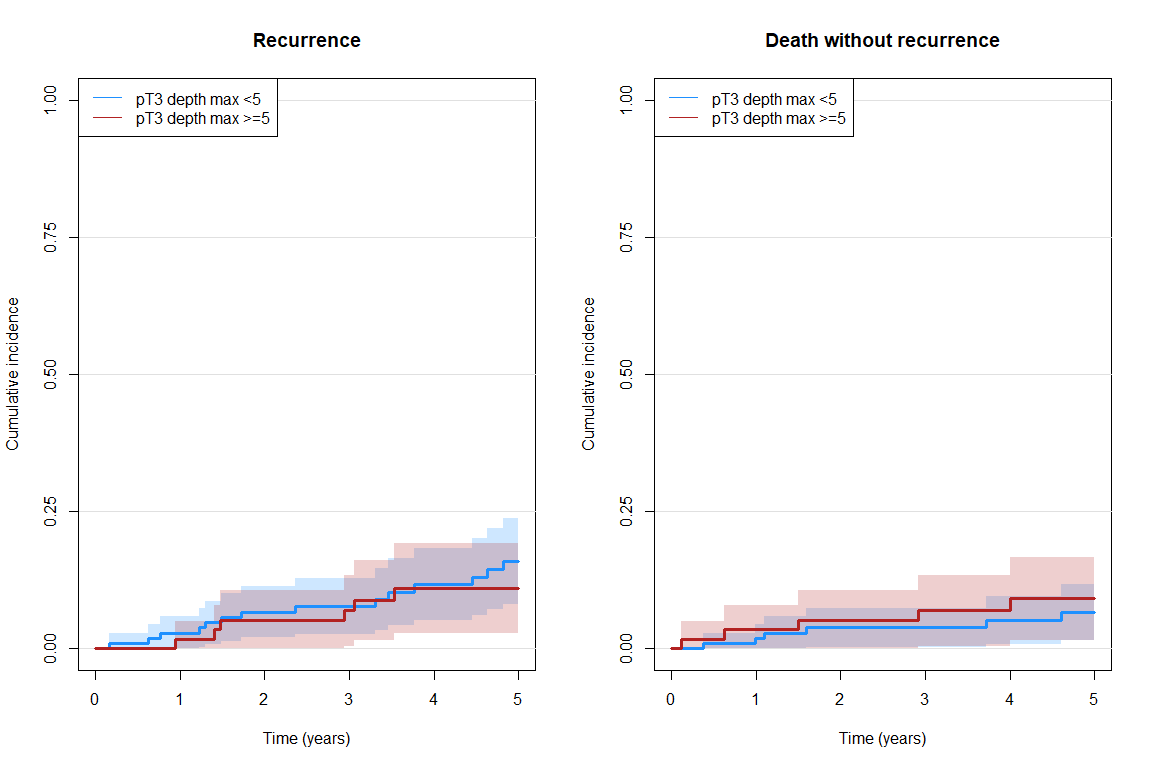


| **Event** | **Time** | **Group** | **Cumulative incidence** | **Lower 95% CI** | **Upper 95% CI** | **P-value** |
| --- | --- | --- | --- | --- | --- | --- |
| Recurrence | 3 years | pT3 <5 | 0.077 | 0.026 | 0.128 | 0.489 |
|  |  | pT3 >=5 | 0.068 | 0.004 | 0.133 |  |
|  | 5 years | pT3 <5 | 0.159 | 0.081 | 0.237 |  |
|  |  | pT3 >=5 | 0.108 | 0.026 | 0.191 |  |
| Death without recurrence | 3 years | pT3 <5 | 0.037 | 0.001 | 0.073 | 0.278 |
|  |  | pT3 >=5 | 0.068 | 0.004 | 0.133 |  |
|  | 5 years | pT3 <5 | 0.065 | 0.014 | 0.117 |  |
|  |  | pT3 >=5 | 0.090 | 0.014 | 0.166 |  |

**Figure 3b. Estimated Recurrence Risk (Cumulative incidence function) in N0 patients, in ELI+ and ELI-.**


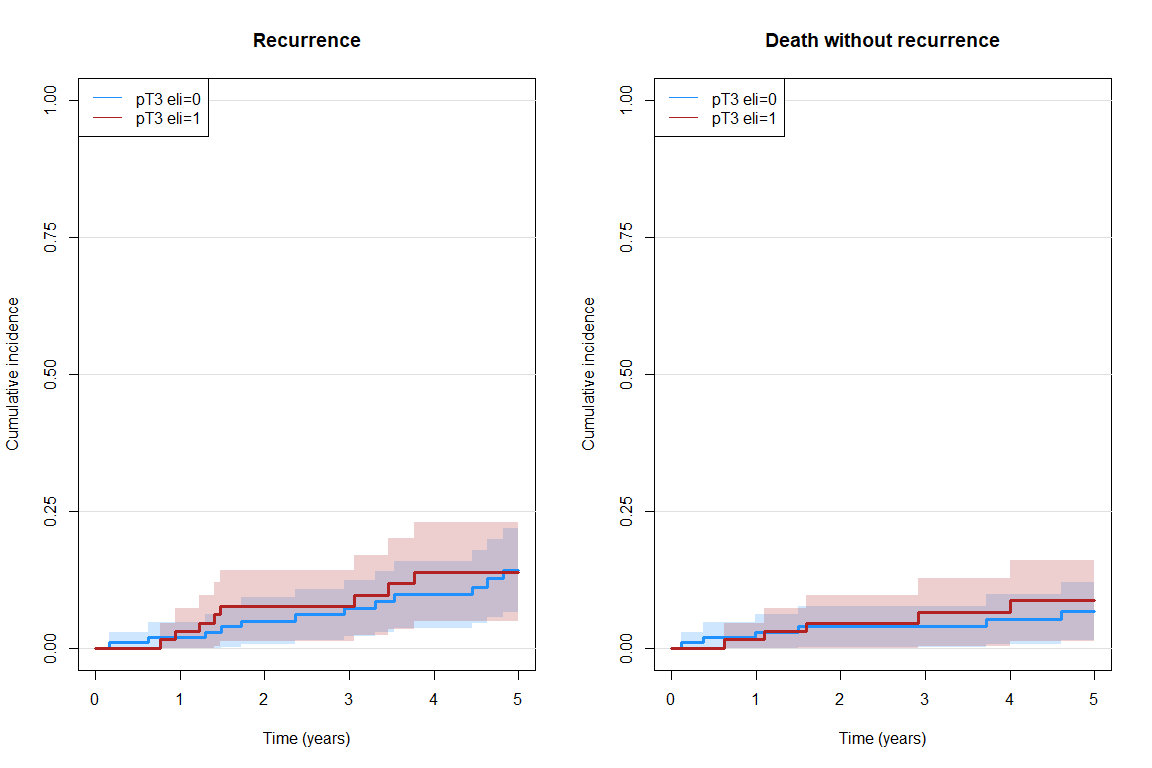


| **Event** | **Time** | **Group** | **Cumulative incidence** | **Lower 95% CI** | **Upper 95% CI** | **P-value** |
| --- | --- | --- | --- | --- | --- | --- |
| Recurrence | 3 years | pT3 eli=0 | 0.072 | 0.021 | 0.124 | 0.885 |
|  |  | pT3 eli=1 | 0.077 | 0.012 | 0.141 |  |
|  | 5 years | pT3 eli=0 | 0.142 | 0.066 | 0.218 |  |
|  |  | pT3 eli=1 | 0.139 | 0.048 | 0.230 |  |
| Death without recurrence | 3 years | pT3 eli=0 | 0.039 | 0.002 | 0.077 | 0.959 |
|  |  | pT3 eli=1 | 0.065 | 0.003 | 0.127 |  |
|  | 5 years | pT3 eli=0 | 0.068 | 0.014 | 0.121 |  |
|  |  | pT3 eli=1 | 0.087 | 0.013 | 0.160 |  |

**Figure 4a.. Kaplan Meier Survival Estimates in pN+ patients for OS in ELI+ and ELI-.**


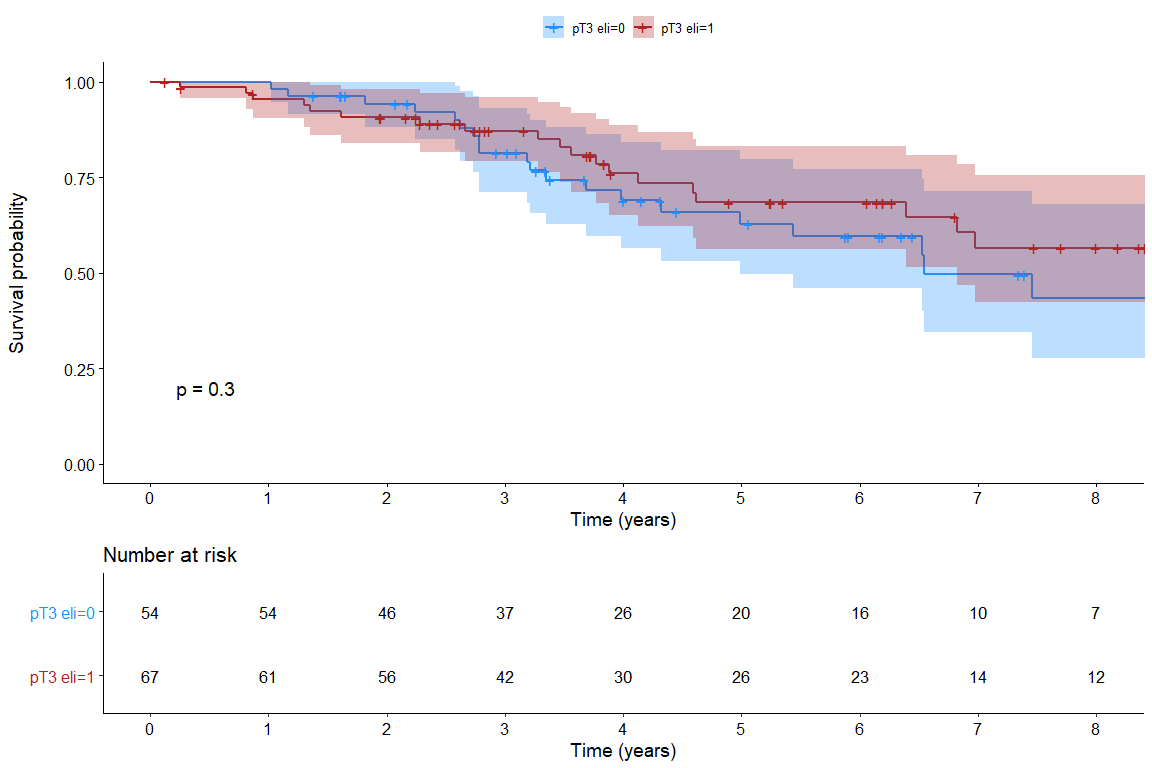


**Figure 4b. Kaplan Meier Survival Estimates in pN+ patients for DFS in ELI+ and ELI-.**
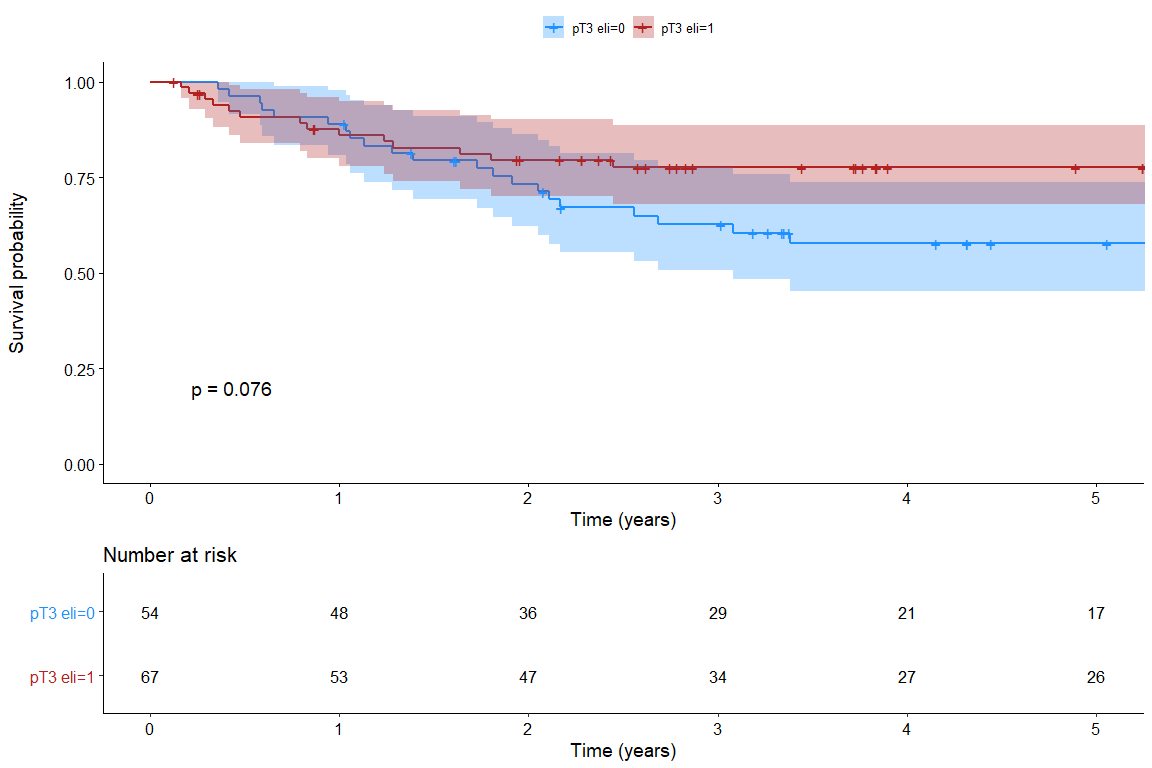


**Figure 5a. Estimated Recurrence Risk (Cumulative incidence function) in N+ patients, in pT3>5mm and pT3<5mm**
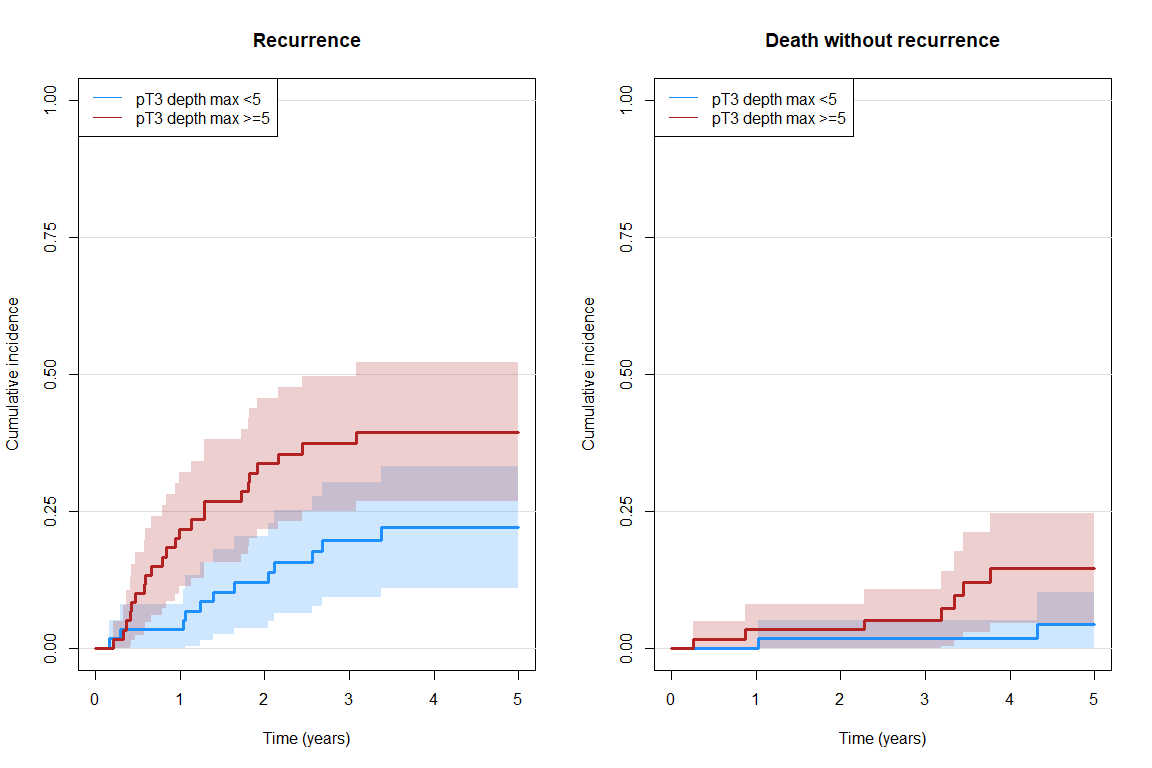


| **Event** | **Time** | **Group** | **Cumulative incidence** | **Lower 95% CI** | **Upper 95% CI** | **P-value** |
| --- | --- | --- | --- | --- | --- | --- |
| Recurrence | 3 years | pT3 <5 | 0.197 | 0.092 | 0.303 | 0.023 |
|  |  | pT3 >=5 | 0.373 | 0.249 | 0.497 |  |
|  | 5 years | pT3 <5 | 0.220 | 0.109 | 0.331 |  |
|  |  | pT3 >=5 | 0.394 | 0.268 | 0.521 |  |
| Death without recurrence | 3 years | pT3 <5 | 0.017 | 0.000 | 0.050 | 0.025 |
|  |  | pT3 >=5 | 0.051 | 0.000 | 0.107 |  |
|  | 5 years | pT3 <5 | 0.042 | 0.000 | 0.101 |  |
|  |  | pT3 >=5 | 0.145 | 0.044 | 0.246 |  |

**Figure 5b. Estimated Recurrence Risk (Cumulative incidence function) in N+ patients, in ELI+ and ELI-**


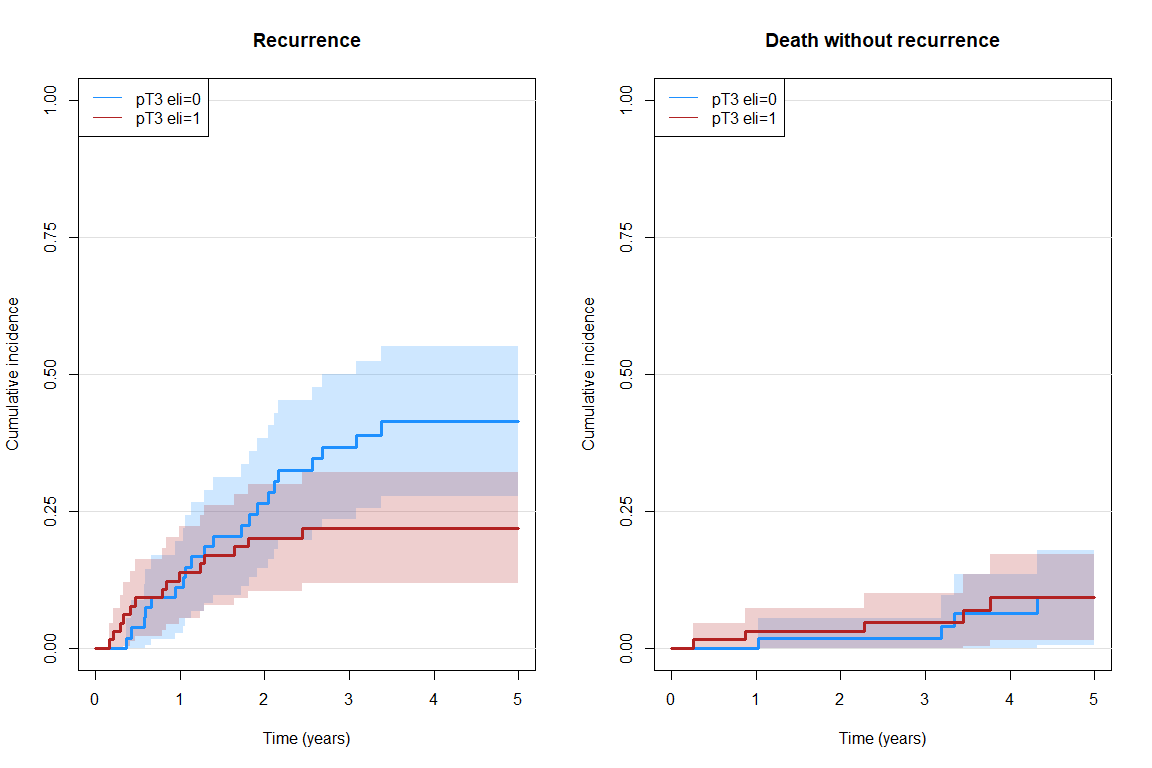


| **Event** | **Time** | **Group** | **Cumulative incidence** | **Lower 95% CI** | **Upper 95% CI** | **P-value** |
| --- | --- | --- | --- | --- | --- | --- |
| Recurrence | 3 years | pT3 eli=0 | 0.367 | 0.235 | 0.500 | 0.069 |
|  |  | pT3 eli=1 | 0.219 | 0.117 | 0.321 |  |
|  | 5 years | pT3 eli=0 | 0.414 | 0.276 | 0.552 |  |
|  |  | pT3 eli=1 | 0.219 | 0.117 | 0.321 |  |
| Death without recurrence | 3 years | pT3 eli=0 | 0.019 | 0.000 | 0.055 | 0.746 |
|  |  | pT3 eli=1 | 0.048 | 0.000 | 0.100 |  |
|  | 5 years | pT3 eli=0 | 0.092 | 0.005 | 0.178 |  |
|  |  | pT3 eli=1 | 0.092 | 0.014 | 0.170 |  |
